# Exploring anti-liver cancer targets and mechanisms of oxyresveratrol: *in silico* and verified findings

**DOI:** 10.1080/21655979.2021.1985328

**Published:** 2021-12-02

**Authors:** Feilan Zhao, Jingru Qin, Yujia Liang, Rui Zhou

**Affiliations:** a Department of Histology and Embryology, Guangxi University of Chinese Medicine, Nanning, Guangxi, PR China; b Department of Pharmacology, College of Pharmacy, Guangxi Medical University, Guangxi, Nanning, PR China; c Department of Hepatobiliary Surgery, Guigang City People’s Hospital, the Eighth Affiliated Hospital of Guangxi Medical University, Guigang, Guangx, PR China

**Keywords:** Oxyresveratrol, liver cancer, bioinformatics, key targets, verification

## Abstract

The aim of current study was to exhume the potential targets and molecular mechanisms of oxyresveratrol, a structurally re-constructed resveratrol, for treating liver cancer through bioinformatics investigation and experimentative validation. To start with, the network pharmacology approach and molecular docking technology were used to uncover all candidate targets of oxyresveratrol to treat liver cancer, accompanied with identified anti-liver cancer targets including estrogen receptor 1 (ESR1), epidermal growth factor receptor (EGFR). In addition, more pharmacological mechanisms of oxyresveratrol against liver cancer were revealed in details. In experimental verification, the clinical samples of liver cancer showed elevated ESR1, EGFR mRNA expressions. The *in-vitro* data indicated that intracellular contents of ESR1, EGFR mRNAs in oxyresveratrol-treated liver cancer cells were reduced. Taken together, the bioinformatics and validated findings have highlighted detailed pharmacological targets and molecular mechanisms of oxyresveratrol for treating liver cancer. Following with experimental verification, the identified genes of ESR1, EGFR may function as potential screening anti-liver cancer markers.

## 1. Introduction

Liver cancer, known as a malignancy in hepatobiliary system, has caused massive deaths around the world, especially in underdeveloped countries or areas [1]. As a common tumor occurred frequently in China, the incidence and mortality in rural areas are still increased yearly due to shortage of early detection and efficient prevention policy [2]. Some Chinese western provinces, such as Guangxi, have found high risk factors for liver cancer induction and development, including hepatitis infection, aflatoxin exposure [3]. Our previous report has suggested parts of Guangxi liver cancer patients with high level of aflatoxin B1 in sera and tumor samples [4]. In addition to surgical excision, clinical prescription using chemotherapy is used to treat different stages of liver cancer patients, such as Fluorouracil, Adriamycin, and Cisplatin [5]. Although targeted drug can be prescribed to control liver cancer, some increasing drug tolerance and poor prognosis are still found after long-term treatment [6]. Compared to clinical medicines, certain compounds isolated from traditional Chinese medicine may potentially use for liver cancer management with low cytotoxicity [7–9]. Pre-clinical data suggest that resveratrol, a well-reported oxidation inhibitor, may reduce excessive oxidative stress and inflammatory damage [10]. Other increasing findings enunciate that resveratrol may function as an anti-cancer agent based on *in vivo* investigation [11,12]. Oxyresveratrol, structurally modified from resveratrol, is a functional compound that has found with effective suppression of tumor cells, such as colorectal cancer [13]. Additional *in vitro* and *in vivo* data demonstrate that oxyresveratrol plays anti-neoplastic effects against liver cancer through inhibiting angiogenesis [14]. However, comprehensive pharmacological mechanisms of oxyresveratrol against liver cancer are still uninvestigated allsidedly. Resoundingly, network pharmacology measurement can roundly uncover detailed targets and molecular pathways of pharmacologically active compounds to treat clinical disorders in preclinical assessment [15,16]. Using this bioinformatics approach, we have identified core targets, biological functions, pathways, and mechanisms of plumbagin action against pancreatic cancer [17]. Taken together, in this study, we designed to employ network pharmacology/molecular docking analyses and experimental verification to identify the key anti-liver cancer targets of oxyresveratrol action before exploring further clinical application.

## 2. Material and methods

### 2.1 Preparation of selected genes in oxyresveratrol and liver cancer

By using Swiss Target Prediction and BATMAN TCM databases, target genes of oxyresveratrol were identified with the ‘Homo sapience’ mode. Furthermore, liver cancer target genes were attained through retrieving in GeneCard, OMIM, Oncodb.hcc databases [18]. All overlapping genes of oxyresveratrol and liver cancer were identified and exhibited by using Venn diagram analysis [19,20].

### 2.2 Establishment of protein–protein interaction (PPI) network

The interaction PPI graph of oxyresveratrol and liver cancer was processed by using STRING (https:// string-db.org/) before identifying crucial targets of oxyresveratrol against liver cancer [21].

### 2.3 Identification of crucial targets in oxyresveratrol against liver cancer

Following the use of Network Analyzer setting-up in Cytoscape v3.7.1 software, degree value in topological parameters, including median and maximum degrees of freedom, was employed to identify all crucial targets. The sieving scopes were designed like that the upper limit was maximum degree value, and the lower limit was twice median degree of freedom [22,23].

### 2.4 Functional and mechanical enrichment analyses of crucial targets

R-language analysis platform was used to provide correlation crucial targets for functional and mechanical annotations, including Gene Ontology (GO) and Kyoto Encyclopedia of Genes and Genomes (KEGG) by using ‘org.Hs.eg.Db’ data packet. Bar charts of enrichment analysis related to crucial targets were identified and plotted according to p-value = 0.05, q-value = 0.05. The signaling pathways in crucial targets were presented and detailed by using ‘pathview’ data packet [24]. In addition, comprehensive network of oxyresveratrol and liver cancer related to overlapping/crucial targets, functions, mechanisms were visualized accordingly [25].

### 2.5 Crucial target molecular docking analysis

The oxyresveratrol molecule was docked with crucial target proteins by using Autodock Vina software, followed with energy range and exhaustiveness as default to gain different binding sites. The active site’s grid box size was designed as previously described [26]. After being docked, the lowest binding energy data, characterized with highest affinity, were screened to display the 3D docking simulation. Root mean square deviation less than 4 Å was the threshold for conformational anastomosis of ligand/proto-ligand [27].

### 2.6 Human sampling

For clinical determination, eight liver cancer advanced patients were enlisted and used for computational validation. These human samples were medically detected as late cancer stage through serial clinical inspections, including imaging test and pathological evaluation. All human cancer or non-cancer samples were resected surgically, and then the samples were used for quantitative PCR analysis. Our clinical procedures used were approved through the hospital ethics committee, and all experiments were carried out according to the principles of Declaration of Helsinki [28,29].

### 2.7 Cultured study in vitro

As described previously [30,31], HepG2, a human liver cancer cell line, weas purchased from Chinese Academy of Sciences (Shanghai, China) and maintained in dulbecco’s modified eagle medium (DMEM) plus 10% fetal calf serum and 1% antibiotics. The cells were treated with dosed oxyresveratrol (purity greater than 98%) at gradient concentrations of 10, 20 μM for lasting 48 hours. For cell proliferation, cell counting kit-8 (CCK8) was used to test oxyresveratrol-treated cells. Meanwhile, the transcript levels of ESR1, EGFR mRNAs were determined through fluorescence quantitative polymerase chain reaction.

### 2.8 Statistical analysis

All statistical data were presented as the means ± standard deviations (SD), and raw data were assessed through statistical product and service solutions 19.0 software (Chicago, IL, USA). All different comparisons were measured using a one-way analysis of variance and Tukey’s post hoc test. The statistical significance was set as *P* value less than 0.05.

## 3. Results

### 3.1 Summarizing of target genes in oxyresveratrol and liver cancer

**Figure 1.**
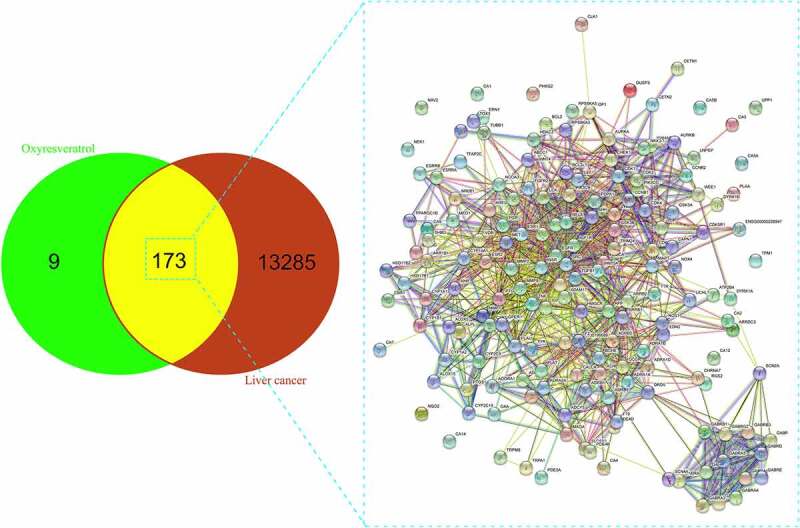
All potential genes, overlapping target proteins of oxyresveratrol and liver cancer identified from respective public databases

A total of 182 oxyresveratrol-associated genes were identified by using different public datasets. And the number of 13,458 liver cancer potential genes was obtained from other the public datasets. As demonstrated in Figure 1, ultimate 173 overlapping genes among oxyresveratrol and liver cancer were harvested and intersected accordingly.

**Figure 2.**
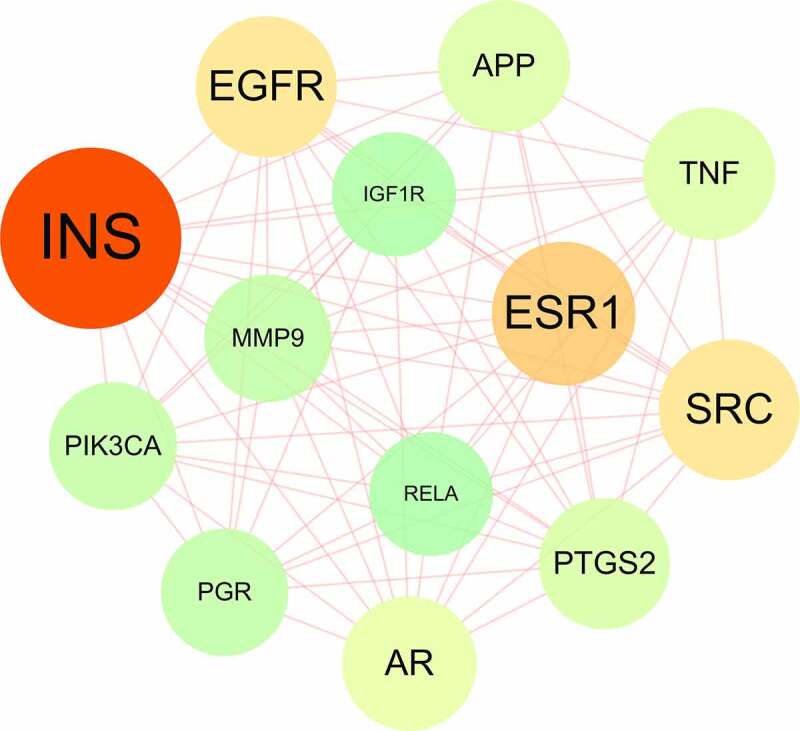
PPI network with 13 crucial targets in oxyresveratrol against liver cancer by using string assay

**Figure 3.**
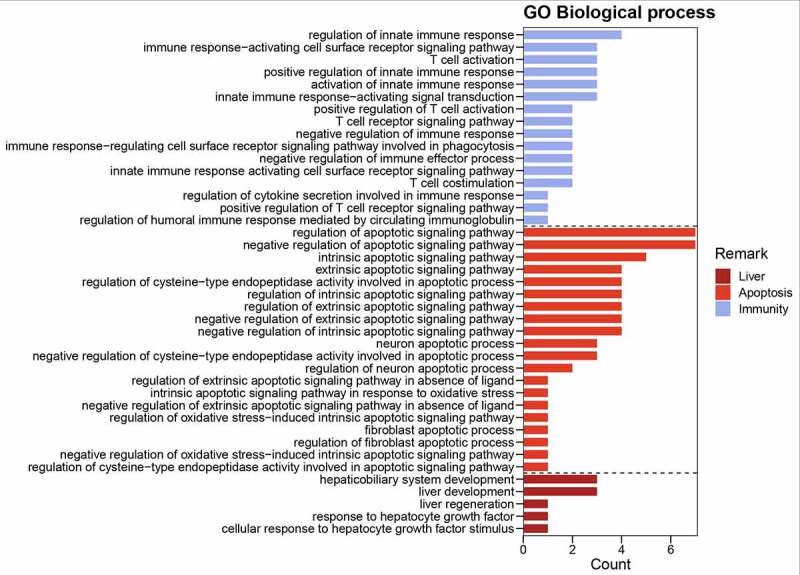
Bar diagram of GO-based biological processes associated with anti-liver cancer functions of oxyresveratrol

**Figure 4.**
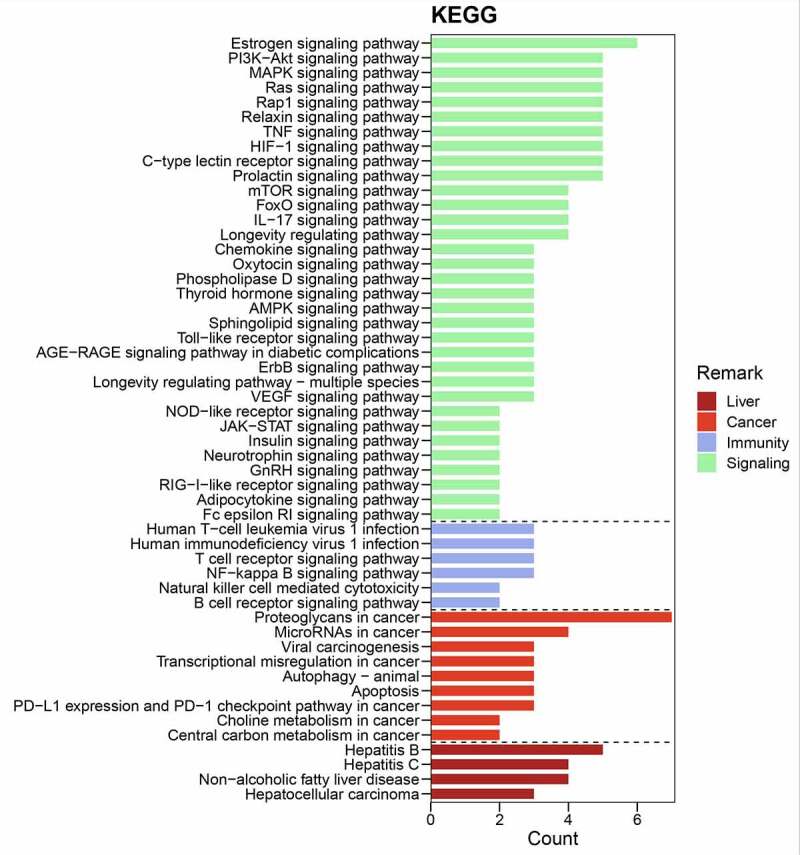
Bar chart of KEGG-based signaling pathways involved in anti-liver cancer mechanisms of oxyresveratrol

**Figure 5.**
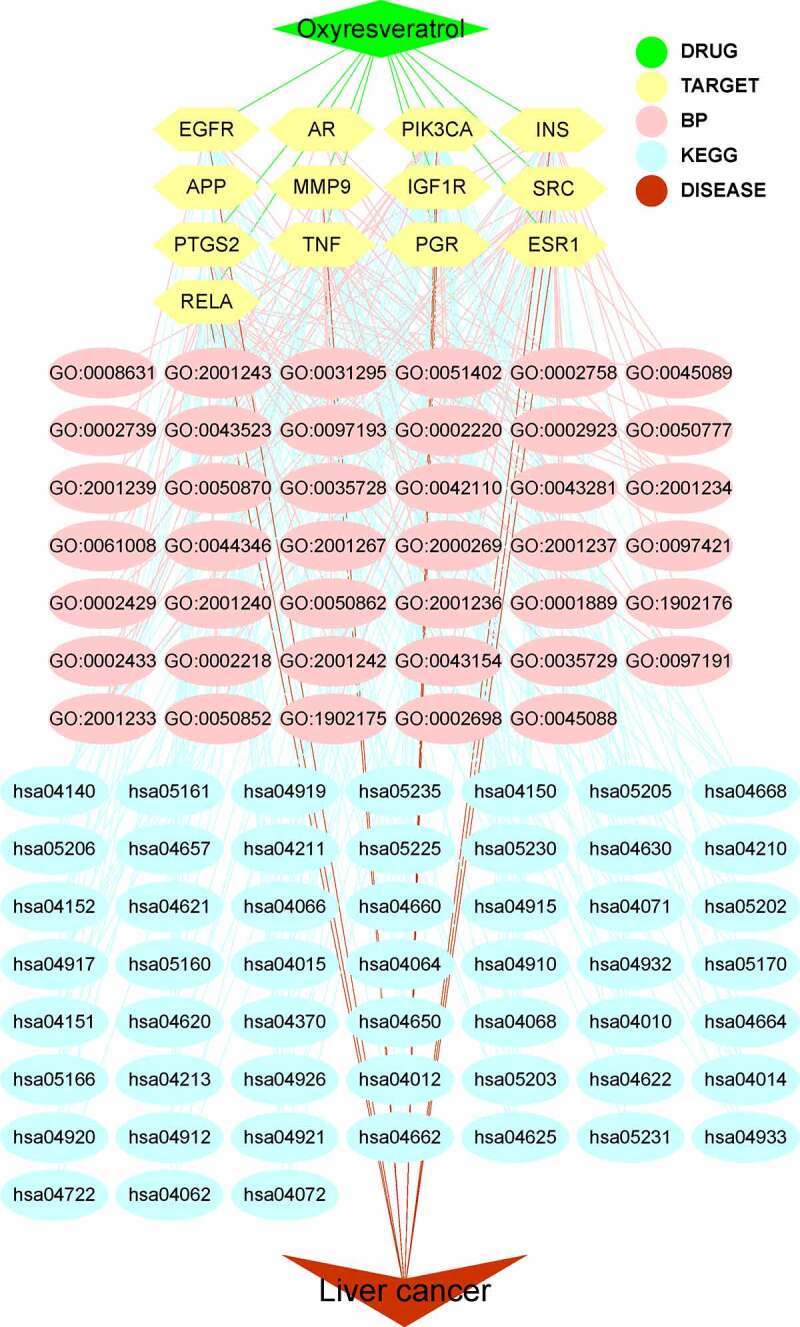
Oxyresveratrol-crucial targets-signaling pathway-liver cancers integrated networks

**Figure 6.**
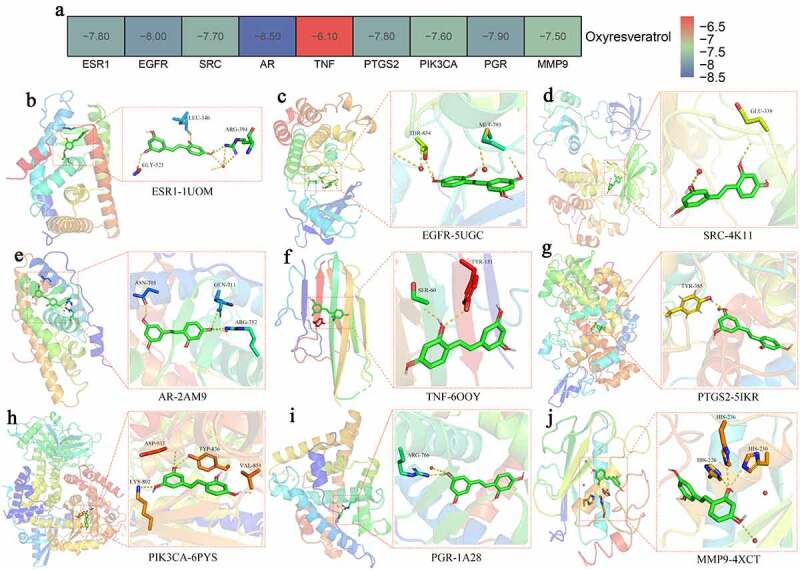
Molecular docking interaction between potent binding capabilities of oxyresveratrol and target proteins. (a) Binding energy parameters; (b) ESR1 (PDB ID: 1UOM); (c) EGFR (PDB ID: 5UGC); (d) SRC (PDB ID: 4K11); (e) AR (PDB ID: 2AM9); (f) TNF (PDB ID: 6OOY); (g) PTGS2 (PDB ID: 5IKR); (h) PIK3CA (PDB ID: 6PYS); (i) PGR (PDB ID: 1A28); (j) MMP9 (PDB ID: 4XCT)

**Figure 7.**
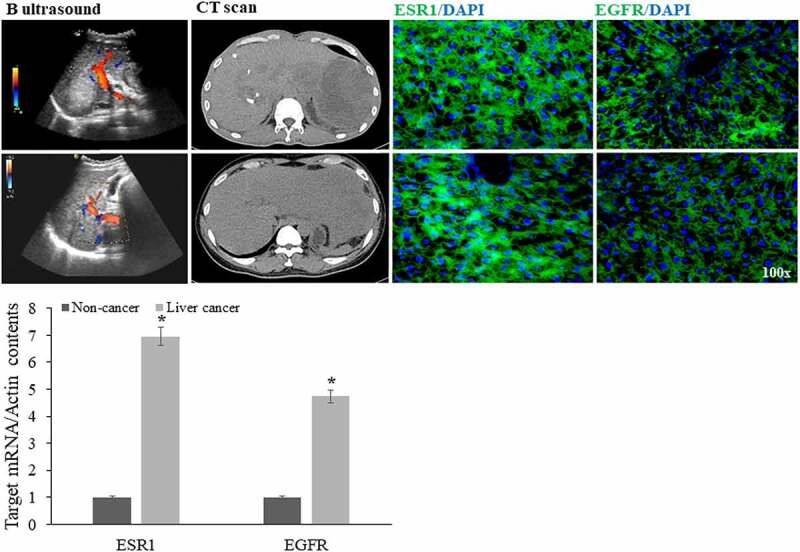
Validated determination in human liver cancer samples based on network pharmacology and molecular docking findings

**Figure 8.**
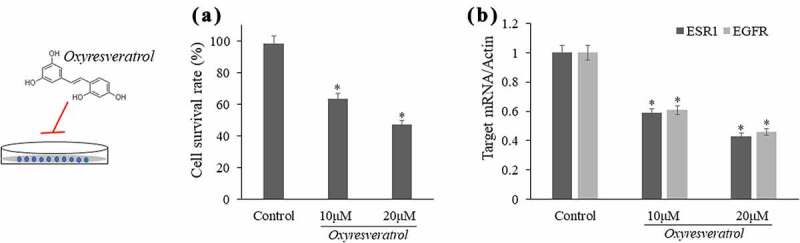
Validated experiments in oxyresveratrol-treated liver cancer cells according to network pharmacology and molecular docking data

### 3.2 Ascertaining of all crucial targets

All 26 crucial targets were selected and identified by topological parameters, in which the screening range was set to 30–83. According to the PPI analysis, 26 crucial targets were connected to each other in network diagram. In brief, the crucial targets included INS, ESR1, EGFR, SRC, AR, TNF, APP, PTGS2, PIK3CA, PGR, MMP9, IGF1R, and RELA (Figure 2).

### 3.3 Functional and mechanical enrichment findings

Figure 3 revealed detailed liver-apoptosis-immunity biological processes or functions in crucial targets. The GO functional annotations indicated that these terms might be potential pharmacological activities against liver cancer. Among KEGG analysis, there are 116 signaling pathways against liver cancer (*P* < 0.05) characterized with significant association. The bar diagram showed that crucial targets associated with main signaling pathways created by count scores, as sorted in Figure 4. More visibly, integrated intersection chart was produced using all current bioinformatics data for connection and visualization (Figure 5).

### 3.4 Molecular docking data

As a result, the binding affinities of oxyresveratrol with ESR1, EGFR, SRC, AR, TNF, PTGS2, PIK3CA, PGR, and MMP9 proteins in liver cancer showed in Figure 6(a). In ESR1 (PDB ID: 1UOM), the binding free energy of oxyresveratrol to protein was −7.8 kcal/mol, in which it formed hydrogen bond with the amino acid residues of LEU-346 (2.6 Å), ARG-394 (3.1 Å), GLY-521 (2.3 Å) Figure 6(b). In EGFR (PDB ID: 5UGC), the binding free energy of oxyresveratrol to protein was −8.0 kcal/mol, in which it formed hydrogen bond with the amino acid residues of MET-793 (2.2 Å) and THR-854 (2.9 Å) (Figure 6c). In SRC (PDB ID: 4K11), the binding free energy of oxyresveratrol to protein was −7.7 kcal/mol, in which it formed hydrogen bond with the amino acid residues of GLU-339 (2.2 Å) (Figure 6d). In AR (PDB ID: 2AM9), the binding free energy of oxyresveratrol to protein was −8.5 kcal/mol, in which it formed hydrogen bond with the amino acid residues of ASN-705 (2.2 Å), GLN-711 (3.1 Å), and ARG-752 (3.0 Å) (Figure 6e). In TNF (PDB ID: 6OOY), the binding free energy of oxyresveratrol to protein was −6.1 kcal/mol, in which it formed hydrogen bond with the amino acid residues of SER-60 (2.2 Å) and TYR-151 (2.7 Å) (figure 6f). In PTGS2 (PDB ID: 5IKR), the binding free energy of oxyresveratrol to protein was −7.8 kcal/mol, in which it formed hydrogen bond with the amino acid residues of TYR-385 (2.0 Å) (Figure 6g). In PIK3CA (PDB ID: 6PYS), the binding free energy of oxyresveratrol to protein was −7.6 kcal/mol, in which it formed hydrogen bond with the amino acid residues of LYS-802 (3.3 Å), TYR-836 (2.8 Å), VAL-851 (2.0 Å), ASP-933 (3.1 Å) (Figure 6h). In PGR (PDB ID: 1A28), the binding free energy of oxyresveratrol to protein was −7.9 kcal/mol, in which it formed hydrogen bond with the amino acid residues of ARG-766 (2.9 Å) (Figure 6i). In MMP9 (PDB ID: 4XCT), the binding free energy of oxyresveratrol to protein was −7.5 kcal/mol in which it formed hydrogen bond with the amino acid residues of HIS-226 (2.8 Å), HIS-230 (3.3 Å), and HIS-236 (3.0 Å) (Figure 6j).

### 3.5 Clinical validation in human samples

Based on current computational findings via molecular docking assay, some human samples of liver cancer and cancer-free were harvested for experimental validation after medically imaging detection, including B ultrasound, computed tomography (CT) scans, and immunostaining positive determination. Other quantitative PCR analysis resulted in elevated expressions of ESR1, EGFR mRNA in cancer samples in comparison with those in non-cancer controls (*P* < 0.05) (Figure 7).

### 3.6 Preclinical validation in oxyresveratrol-treated cells

The experimental evidences *in vitro* showed that oxyresveratrol-treated HepG2 cells resulted in lowered cell proliferation when compared to that in vehicle control (*P* < 0.05) (Figure 8a). In quantitative analysis using PCR, the data showed that down-regulated transcript expressions of ESR1, EGFR mRNAs in oxyresveratrol-treated cells were observed (*P* < 0.05) in a dose-related manner (Figure 8b).

## 4. Discussion

Current bioinformatics networking assay illuminated that the pharmacological effectiveness of oxyresveratrol against liver cancer was chiefly implicated in crucial target-associated signaling pathways, such as apoptosis, autophagy, NF-kappa B signaling pathway, T-cell receptor signaling pathway, natural killer cell-mediated cytotoxicity, estrogen signaling pathway. The estrogen signaling pathway is a key pathway that can function in cell proliferation, apoptosis, invasion, and angiogenesis. Additionally, the estrogen signaling pathway regulates the expression of functional genes that maintain normal cell function, cancer cell growth and metastasis [32,33]. Notably, current network pharmacology analysis has identified that some crucial targets are mostly related to the estrogen signaling pathway.

Our bioinformatics findings effectively and completely identify all pharmacological targets of oxyresveratrol against liver cancer. By using molecular docking analysis, oxyresveratrol highlighted potent binding affinity accompanied with the high docking scores in crucial proteins, including ESR1, EGFR. Thus, some of *in silico* findings would be validated via experimental tests. ESR1, a steroid receptor, plays an important role in regulating tumorigenesis, such as breast cancer [34]. If mutation, ESR1 pre-exist in primary malignancy and it can be activated during metastasis. Thus, suppression of ESR1 gene activation may be used for anti-cancer strategy. EGFR is the receptor of epithelial growth factor that is responsible for cell proliferation and signal transduction [35]. Other findings indicated that exosome-transferred EGFR modulates liver microenvironment to trigger metastatic gastric cancer development [36]. Therefore, the EGFR may be another potential anti-cancer target. In current validated experiments, human liver cancer samples showed upregulated ESR1, EGFR mRNA expressions. Following the oxyresveratrol treatments *in vitro*, the intracellular mRNAs of ESR1, EGFR were down-regulated. These validated findings demonstrated that the ESR1, EGFR can be anti-liver cancer pharmacological targets exerted by oxyresveratrol. It is reasoned that these bioinformatics outcomes suggest the oxyresveratrol monotherapy or combination with another existing medicine for treating liver cancer. As limited, current bioinformatics findings need to be in-depth validated clinically before the use of oxyresveratrol treating liver cancer in future clinical practice.

## 5 Conclusion

This bioinformatics report indicates that oxyresveratrol is the potent bioactive compound against liver cancer. In addition, current findings provide the revealment of detailed anti-liver cancer biotargets and molecular mechanisms of oxyresveratrol, possibly through controlling the key signaling pathway against liver cancer cells.
